# Disseminated nocardiosis in a patient with nephrotic syndrome following HIV infection

**DOI:** 10.3892/etm.2014.1883

**Published:** 2014-08-06

**Authors:** LETIAN ZHOU, HONG LIU, FANG YUAN, SHUGUANG YUAN

**Affiliations:** Department of Nephrology, The Second Xiangya Hospital, Research Institute of Nephrology, Central South University, Changsha, Hunan 410011, P.R. China

**Keywords:** nocardiosis, nephrotic syndrome, human immunodeficiency virus

## Abstract

In the present study, a case of disseminated abscesses caused by *Nocardia* in a patient undergoing immunosuppressive therapy for nephrotic syndrome and infected with human immunodeficiency virus (HIV) is described. To the best of our knowledge, this is the first such case to be reported. The patient had membranous nephropathy and received systemic corticosteroid therapy for one year. During this time, the patient was diagnosed with HIV and developed disseminated abscesses in the lungs, brain and hip. Pathogens isolated from sputum and pus were identified as *Nocardia asteroides*. The patient was successfully treated following surgical drainage of the abscesses and by oral administration of trimethoprim-sulfamethoxazole.

## Introduction

Nocardiosis is a rare but severe pyogenic infection that is most commonly found in patients who are immunocompromised (1). Pulmonary disease is the most common presentation in patients with nocardiosis and approximately one-third of such patients have a disseminated disease (2). Common predisposing factors for nocardial infection include corticosteroid therapy, chemotherapy for neoplasms and acquired immune deficiency syndrome. Patients with nephrotic syndrome also have high morbidity of nocardial infection due to immunosuppressive regimens. Hwang *et al* (3) previously described a patient with nephrotic syndrome, accompanied by pulmonary nocardiosis, but negative for human immunodeficiency virus (HIV); however, to the best of our knowledge, a patient with nephrotic syndrome with nocardiosis who is also HIV-positive has yet to be reported. In the present study, an unusual case of a patient with nephrotic syndrome who developed disseminated nocardiosis following immunocompromised therapy and HIV infection is described.

## Case report

**Figure 1:**
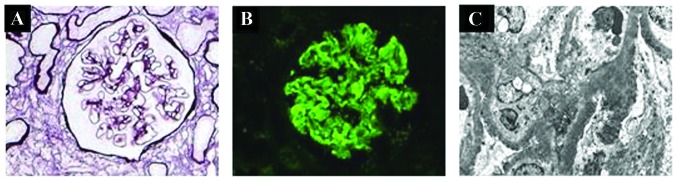
Renal biopsy obtained from the patient. (A) A periodic acid-silver methenamine (PAM)-stained paraffin-embedded section shows thickened glomerular basement membrane (magnification, ×400). (B) Immunofluorescence shows moderate granular staining for immunoglobulin G (IgG) along the capillary wall (magnification, ×400). (C) An electron micrograph shows marked subepithelial electron-dense deposits (magnification, ×2,000).

During the autumn of 2010, a 55-year-old male developed edema in his lower legs and foamy urine. The patient was admitted to the Changde Hospital, (Changde, China) and was diagnosed with nephrotic syndrome. A test for antibodies against HIV was found to be negative. The patient was treated with prednisone therapy (60 mg/day) for 3 months, and the dosage was then reduced to 5 mg/month. However, the patient failed to improve after 10 months and was transferred to the Second Xiangya Hospital (Changsha, China) for further investigation and treatment. On admission, bilateral lower extremity edema was observed. Laboratory studies identified a persistent albuminuria with an initial total 24 h urinary protein loss of 9.6 g and a serum albumin level of 13.4 g/l. The creatinine clearance rate was 58 ml/min. The blood urea nitrogen level was normal; however, the serum cholesterol level was grossly elevated (7.32 mmol/l). The patient tested negative for antibodies against HIV. Renal biopsy showed membranous nephropathy (Fig. 1) and the patient was administered 30 mg prednisone daily and 1 mg tacrolimus daily.

**Figure 2:**
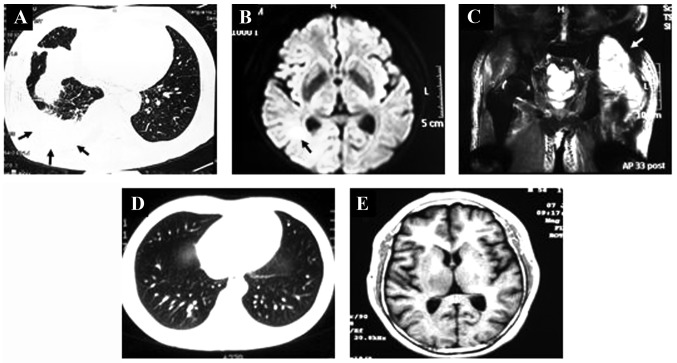
MRI and CT scans results prior to and following treatment with trimethoprim-sulfamethoxazole. (A) CT scan shows abscesses in the right lung prior to treatment. MRI scans reveal (B) an abscess in the right brain, as well as (C) a large abscess in the left gluteal region prior to treatment. (D) CT scan shows that the abscess in the right lung disappeared following treatment. (E) MRI shows that the abscess in the right brain disappeared following treatment. MRI, magnetic resonance imaging; CT, computed tomography.

After ~3 months of treatment, the 24 h urinary protein loss was reduced to 4.8 g/day and the level of serum albumin rose to 20.2 g/l. However, the patient developed a fever and started coughing up purulent sputum. The patient was diagnosed with pneumonia in Hanshou Hospital (Hanshou, China) and was treated with various combinations of penicillin, ceftriaxone and itraconazole. However, the patient’s temperature continued to increase and he was transferred again to the Second Xiangya Hospital. On admission, the body temperature of the patient was 39.2°C. Inspiratory moist rales were audible in the right inferior lung. A 2-cm hard subcutaneous nodule was palpable at the right lower abdomen. The white blood cell count was 19.7×10^9^/l with 85% neutrophils. Tests for antibodies against tuberculosis were negative and no *Mycobacterium tuberculosis* was found in the sputum and hydrothorax. A chest computed tomography (CT) scan revealed a nodule in the right lower lung field with pleural effusion (Fig. 2A). After 1 week, the patient developed left hip pain and magnetic resonance imaging (MRI) revealed a brain abscess in the right temporal occipital junction (Fig. 2B), as well as a larger abscess in the left gluteal region (Fig. 2C). Pathogens isolated from sputum and pus from the subcutaneous abdomen were identified as *Nocardia asteroides*. Other pathogens, including bacteria, mycobacteria and fungi were not isolated. The patient was therefore diagnosed with nocardiosis. Antibodies against HIV were tested for again and this time were found to be positive. The patient was treated with surgical drainage of the hip abscess and the oral administration of trimethoprim-sulfamethoxazole (0.96/4.8 g/day). The patient was discharged after 50 days of hospitalization. The trimethoprim-sulfamethoxazole therapy was continued and the patient remained in a satisfactory condition. The results from the CT and MIR scans showed that the size of the lung (Fig. 2D) and brain (Fig. 2E) abscesses decreased gradually after 3 months and disappeared completely after 6 months.

## Discussion

To the best of our knowledge, disseminated nocardiosis in a patient with nephrotic syndrome and HIV infection has not been previously reported. *Nocardia* species are ubiquitous environmental microorganisms that are present worldwide and belong to a diverse group of bacteria known as aerobic actinomycetes. So far >50 species of the genus *Nocardia* have been characterized, with ≥16 species that have been implicated in human infection (4). The most common of these include *Nocardia asteroides*, *Nocardia brasiliensis* and *Nocardia farcinica*. In most cases, *Nocardia* is an opportunistic pathogen, with the majority of infections occurring in immunocompromised hosts, including those with long-term corticosteroid exposure, malignancy, HIV infection or a history of transplantation.

Peleg *et al* (2) previously demonstrated that treatment with high doses of prednisone, a history of CMV infection and an elevated mean calcineurin inhibitor level are independent risk factors for *Nocardia* infection in organ transplant recipients. Patients with nephrotic syndromes also have high morbidity rates due to nocardial infection as a result of immunosuppressive regimens. Particular attention should be given to nocardial infection in immunocompromised patients if the infection is not controlled following treatment with several antibiotics. In the present study, the patient had two of these three risk factors: previous treatment with prednisone and elevated levels of calcineurin inhibitor. Tacrolimus, a calcineurin inhibitor, inhibits T-cell activation by binding to FK-binding protein 12. T cells are essential for an adequate host response against *Nocardia* infection, primarily through the activation of macrophages and the stimulation of a cellular immune response (5).

In addition, in the present study the patient was infected with HIV. Prior to the renal biopsy, the patient had already been administered high-doses of prednisone for 10 months without infection. However, 3 months following treatment with prednisone and tacrolimus, the patient developed nocardiosis, an opportunistic pathogen infection. The occurrence of an opportunistic infection following the reduction of the steroid dose is uncommon. Therefore, an HIV test was performed again and the patient was found to have acquired an HIV infection during these 3 months. The HIV infection further destroyed the immune system of the patient and induced the nocardial infection. The results from the present study indicate that in cases of opportunistic infections, further investigation into the risk factors of the patient is required.
